# Dynamic Changes in Spectral and Spatial Signatures of High Frequency Oscillations in Rat Hippocampi during Epileptogenesis in Acute and Chronic Stages

**DOI:** 10.3389/fneur.2016.00204

**Published:** 2016-11-28

**Authors:** Pan-Pan Song, Jing Xiang, Li Jiang, Heng-Sheng Chen, Ben-Ke Liu, Yue Hu

**Affiliations:** ^1^Department of Neurology, Children’s Hospital of Chongqing Medical University, Chongqing, China; ^2^Ministry of Education Key Laboratory of Child Development and Disorders, Chongqing, China; ^3^Chongqing Key Laboratory of Pediatrics, Chongqing, China; ^4^China International Science and Technology Cooperation Base of Child Development and Critical Disorders, Chongqing, China; ^5^Department of Neurology, MEG Center, Cincinnati Children’s Hospital Medical Center, Cincinnati, OH, USA

**Keywords:** high frequency oscillations, epileptogenesis, qualitative analysis, quantitative analysis, spectral power

## Abstract

**Objective:**

To analyze spectral and spatial signatures of high frequency oscillations (HFOs), which include ripples and fast ripples (FRs, >200 Hz) by quantitatively assessing average and peak spectral power in a rat model of different stages of epileptogenesis.

**Methods:**

The lithium–pilocarpine model of temporal lobe epilepsy was used. The acute phase of epilepsy was assessed by recording intracranial electroencephalography (EEG) activity for 1 day after status epilepticus (SE). The chronic phase of epilepsy, including spontaneous recurrent seizures (SRSs), was assessed by recording EEG activity for 28 days after SE. Average and peak spectral power of five frequency bands of EEG signals in CA1, CA3, and DG regions of the hippocampus were analyzed with wavelet and digital filter.

**Results:**

FRs occurred in the hippocampus in the animal model. Significant dynamic changes in the spectral power of FRS were identified in CA1 and CA3. The average spectral power of ripples increased at 20 min before SE (*p* < 0.05), peaked at 10 min before diazepam injection. It decreased at 10 min after diazepam (*p* < 0.05) and returned to baseline after 1 h. The average spectral power of FRs increased at 30 min before SE (*p* < 0.05) and peaked at 10 min before diazepam. It decreased at 10 min after diazepam (*p* < 0.05) and returned to baseline at 2 h after injection. The dynamic changes were similar between average and peak spectral power of FRs. Average and peak spectral power of both ripples and FRs in the chronic phase showed a gradual downward trend compared with normal rats 14 days after SE.

**Significance:**

The spectral power of HFOs may be utilized to distinguish between normal and pathologic HFOs. Ictal average and peak spectral power of FRs were two parameters for predicting acute epileptic seizures, which could be used as a new quantitative biomarker and early warning marker of seizure. Changes in interictal HFOs power in the hippocampus at the chronic stage may be not related to seizure occurrence.

## Introduction

There is increasing evidence that the brain generates electromagnetic signals over a very wide frequency range. High frequency brain signals (HFBS, >70 Hz) are also called high frequency oscillations (HFOs), which typically include ripples and fast ripples (FRs) (1, 2). The highest frequency components identified in brain are approximately 2,500–2,800 Hz (3). Median frequency brain signals (MFBS) in a frequency range of 1–70 Hz are currently used in clinical practice (4). One of the major clinical applications of measuring brain electromagnetic signals is diagnosis of epilepsy. Epileptic spikes (14–70 Hz) recorded by electroencephalography (EEG) are the hallmark for diagnosis (5, 6). However, not every epilepsy patient has spikes during an EEG recording. Therefore, researchers have been investigating new epilepsy biomarkers (7, 8). Recent reports have shown that HFBS show promise as new biomarkers for epilepsy (8, 9). HFBS also closely correlate with seizure severity (10) and are highly localized to the epileptogenic or ictogenic zones (7, 8, 11). Therefore, HFBS may play a key role in epileptogenesis and epileptogenicity (12).

Pilocarpine is a postganglionic cholinergic drug that can directly excite M-cholinergic receptors to produce quasi-cholinergic effects (13). It can be used to activate central cholinergic receptors to induce seizures. The rat pilocarpine model of temporal lobe epilepsy (TLE) can be divided into three phases (13): (i) an acute phase, which includes the duration of status epilepticus (SE) and 6–24 h after SE; (ii) a latent phase lasting approximately 7–42 days after SE, during which no organized activity is recorded and EEG is usually normal; and (iii) a chronic phase, characterized by spontaneous recurrent seizures (SRSs). The model of TLE induced by pilocarpine in rats, which reproduces most clinical and neuropathological features of human TLE, includes SRSs after SE. Therefore, this model is the most commonly used animal model of TLE (14). The cerebral mechanisms of HFOs during seizures have been investigated with animal models (15, 16). Two types of HFOs have been found in epileptic rats after kainic acid injection; one in the range of 100–200 Hz and the other in the range of 200–500 Hz (16).

The objective of the present study was to analyze the spectral and spatial signatures of HFOs in the hippocampus in the rat pilocarpine model of TLE during epileptogenesis in the acute and chronic stages. We quantified average spectral power and the peak spectral power of HFOs. We hypothesized that elevation of HFO spectral power was a parametric biomarker of epileptogenesis, which could be used to quantify initiation and propagation of seizures. The major innovation of the present study is the unification of spectral and spatial signatures of HFOs to quantify epileptic abnormality. We consider this study is important, because it sheds light on the role of HFOs during seizures and lays a foundation for using HFOs as biomarkers for developing new anti-epileptic drugs in the future.

## Materials and Methods

### Animals and Surgery

All experiments were performed according to the experimental guidelines of Chongqing Medical University, and the protocol was approved by the ethics committee of Chongqing Medical University. Adult male Sprague-Dawley rats (200 ± 20 g) were obtained from the Animal Research Institute of Chongqing Medical University. The rats were injected intraperitoneally (IP) with penicillin (1 ml/kg, 160,000 U/ml) 30 min before operation to prevent intracranial infection. They were then anesthetized with 10% chloral hydrate (3 ml/kg, IP) and positioned in a stereotaxic frame (Shenzhen Reward Life Science Company), so that lambda and Bregma lay in the same horizontal plane. The top of the rat’s head was shaved and chemically sterilized with iodine. A midsagittal incision was made in the skin to expose bregma and the lambdoidal suture, and 30% hydrogen peroxide solution was applied to remove excess soft tissue from the skull. Coordinates for areas of interest in the hippocampus were as follows: CA1 – AP −3.3 to 3.7 mm from Bregma, ML 2.0–3.0 mm, and DV 3.0–3.5 mm from the surface of neocortex (17); CA3 – AP −3.3 mm, ML 3.5–3.7 mm, DV 3.0–3.5 mm (17), DG – AP −5.6 mm, ML 4.0 mm, and DV 6.0 mm (18, 19). The reference electrode was located in the surface of neocortex of the bilateral parietal lobe (AP 7.0 mm; ML 6.0 mm). We confirmed these coordinates by Nissl staining. The left forehead was used as a ground (AP 2.0 mm; ML 2.0 mm). Holes were drilled in the corresponding position on the skull, and an implanted microelectrode (nichrome wires, 0.1 mm in diameter) was inserted and fastened to the skull with dental cement (Shanghai medical equipment Limited by Share Ltd. dental material factory). Postoperatively, rats were fed separately and maintained on a 12-h light/dark cycle (lights on at 08:00 h) at a controlled ambient temperature (22–25°C). Food and water were available *ad libitum*.

### Epileptic Model and Electroencephalographic Recordings

Beginning 3 days after surgery, wide band recordings of electrical activity were performed for 5–8 h every day until the day of lithium–pilocarpine treatment (9 days after surgery). EEGs were recorded in freely moving rats using a digital acquisition system (EEG 1200 systems, 32 channels, Nihon-Kohden Corporation, Tokyo, Japan), while at the same time a synchronous video monitoring system was used to detect behavioral seizures. Video monitoring was then continued while rats were sitting in their home cages. Physiological data were magnified once by a preamplifier, 1,000 times by a differential amplifier, and were recorded wide band and sampled at 1 kHz.

On day 9 after surgery, rats were injected with pilocarpine (50 mg/kg IP, Sigma, Canada) 18–20 h after an injection of lithium chloride (127 mg/kg IP, Sigma). If generalized seizures (stage 4 of Racine’s criterion) (20) were not elicited within 30 min, a second injection of pilocarpine (10 mg/kg, IP) was administered. SE was defined as the exhibition of continuously generalized seizures for at least 60 min without returning to normal behavior between seizures. Atropine sulfate (1 mg/kg IP, Shanghai and Feng pharmaceutical companies) was injected to limit peripheral cholinergic effects 10 min after injection of pilocarpine. SE was arrested using diazepam (10 mg/kg IP, Shanghai Asahi Dongpu Pharmaceutical Co. Ltd).

Electroencephalographys were recorded continuously for at least 3.5 h after diazepam injection. Glucose saline (2 ml/day for 3 days, 1 ml 10% glucose + 1 ml 0.9% sterile saline) was injected IP to reduce mortality after SE. Starting 24 h after SE, EEG activity was recorded for 5–8 h/day (16, 18) for 28 consecutive days. Additionally, rats continued to be monitored for behavioral seizures, and the number of SRSs was recorded.

To minimize individual variation, we used a self-controlled study (e.g., before and after seizures or before and after injection) to analyze the dynamic changes in HFOs spectral power during epileptogenesis in the acute and chronic stages. In our study, 15 rats were successfully induced with SE. Due to the disturbance of the implanted electrodes, only 9 of 15 rats were recorded for 28 consecutive days after SE successfully. These animals were divided into two groups: an acute epilepsy group and a chronic epilepsy group. In the acute epilepsy group (*n* = 15), each animal was recorded in the acute phases of the epileptic model, with recordings performed consecutively on the first day after SE. In the chronic epilepsy group (*n* = 9), EEG activity was recorded for 28 consecutive days after SE to characterize the latent and chronic phases of the epileptic model.

### Electroencephalographic Analysis

In the acute phase of the epileptic model, 10 min samples of electrical activity were selected for further analysis at eleven time points (1 day before SE; 30, 20, and 10 min before SE; 10 min after SE; 10 min before diazepam; 10 min after diazepam; 1, 2, and 3 h after diazepam; 1 day after SE).

In chronic phases of epileptic model, three 10 min samples of electrical activity were selected for further analysis at six time points (1 day before SE; 1, 3, 7, 14, and 28 days after SE). All samples were taken during periods of waking, with small movement disturbance and at selected samples interval of at least 1 h.

Analysis of behavioral and electrophysiological data was performed by three individuals who were blinded to the goal of the experiments. For counting behavioral seizures between recording sessions, videotapes were reviewed and detected seizures were scored on the basis of Racine’s scale (20). Raw EEG signals were analyzed by qualitative and quantitative methods. Qualitative analysis used db5 wavelet in-house software designed in MATLAB, which decomposed the EEG signal into five layers and extracted five frequency bands spanning 16.5–31.25, 31.25–62.5, 62.5–125, 125–250, and 250–500 Hz [(fs/2)/2*^n^*]. Quantitative analysis used time–frequency analysis to extract three frequency bands spanning 40–80 Hz (γ oscillations), 80–200 Hz (Ripples), and 200–500Hz (FRs) and to carry out average spectral power and peak spectral power analysis.

To detect HFOs, we used wavelet transforms to decompose time domain waveforms to time–frequency domain spectral data. We used Morlet wavelet algorithms because brain activity is non-stationary and the wavelet is well suited for non-stationary data (21, 22). From a mathematic point of view, Morlet wavelets have a Gaussian window shape in both time and frequency, while maintaining a sinusoidal underlying structure. This wavelet structure yields easily interpretable results in time and frequency domains because they yield qualitatively similar data as when a time–frequency analysis is done with a Fourier transform. Since brain activation in a given time window might be in different frequency ranges with different amplitudes, we used a different sigma value for each frequency to capture the time–frequency changes. In addition, a correction was necessary to make the time–frequency data zero-mean. Consequently, the following equation was used in our study:

(1) $$g(t,f)=c_{\sigma }\pi^{-\frac{1}{4}}e^{-\frac{1}{2}t^{2}}(e^{i\sigma t}-\kappa_{\sigma })$$

In the equation, *t* indicates time, *f* indicates a specific frequency; κ_σ_ indicates the admissibility, and *c*_σ_ indicates a normalized constant for frequency *f*. *g*(*t, f*) indicates wavelet coefficients for a given frequency bin. If signals appeared in the given sensitive time (a small sigma value) and sensitive frequency (a large sigma value) ranges, they were enhanced (21, 22).

To precisely detect high frequency signals, we used accumulated spectrogram to quantify brain signals (21, 22). Accumulated spectrogram has made a few changes to the conventional wavelet transform. First, our wavelet transform for each frequency band had a dynamic sigma value, so we could enhance the sensitivity of our wavelet transform to frequency in high frequency neuromagnetic signals. Second, the time–frequency representations of EEG signals were normalized according to the magnitude of each frequency bin across all EEG channels to ensure all frequency bins contributed equally to the quantification. This compensates for the fact that high frequency signals are typically obscured by low frequency signals. Third, the frequency resolution (number of frequency bins) in our time–frequency transform could be theoretically increased or decreased to infinity. This is important because the exact frequency signatures of EEG signals vary among brain areas (23).

### Statistical Analysis

Statistical analysis was performed using the SPSS 17.0 statistical software package. Quantitative data are expressed as mean ± SD. The comparison of k-related samples used the Friedman test, and two related samples were compared using the Wilcoxon signed-rank sum test. Statistical significance was set at *p* < 0.05.

## Results

### Behavioral Study

There were no abnormal behaviors observed during the 18–24 h following injection with lithium chloride. All rats presented with peripheral cholinergic effects after injection with pilocarpine, including pupil narrowing, piloerection, weeping blood, diarrhea, and wet dog shake (WDS). It took approximately 14–68 min (average 33.13 ± 15.01 min) for the animals to reach stage 4–5 of Racine’s criterion. Peripheral cholinergic effects gradually disappeared after injection with atropine. SE disappeared after injecting diazepam. After a latent phase lasting approximately 3–10 days (6 ± 4.82 days), during which no organized activity was recorded, rats that developed chronic epilepsy had repeated SRSs that reached stages 1–5 of Racine’s criterion. The number of SRSs of chronic epileptic models was 56.22 ± 12.15 within 4 weeks.

### Qualitative Analysis of HFOs

**Figure 1:**
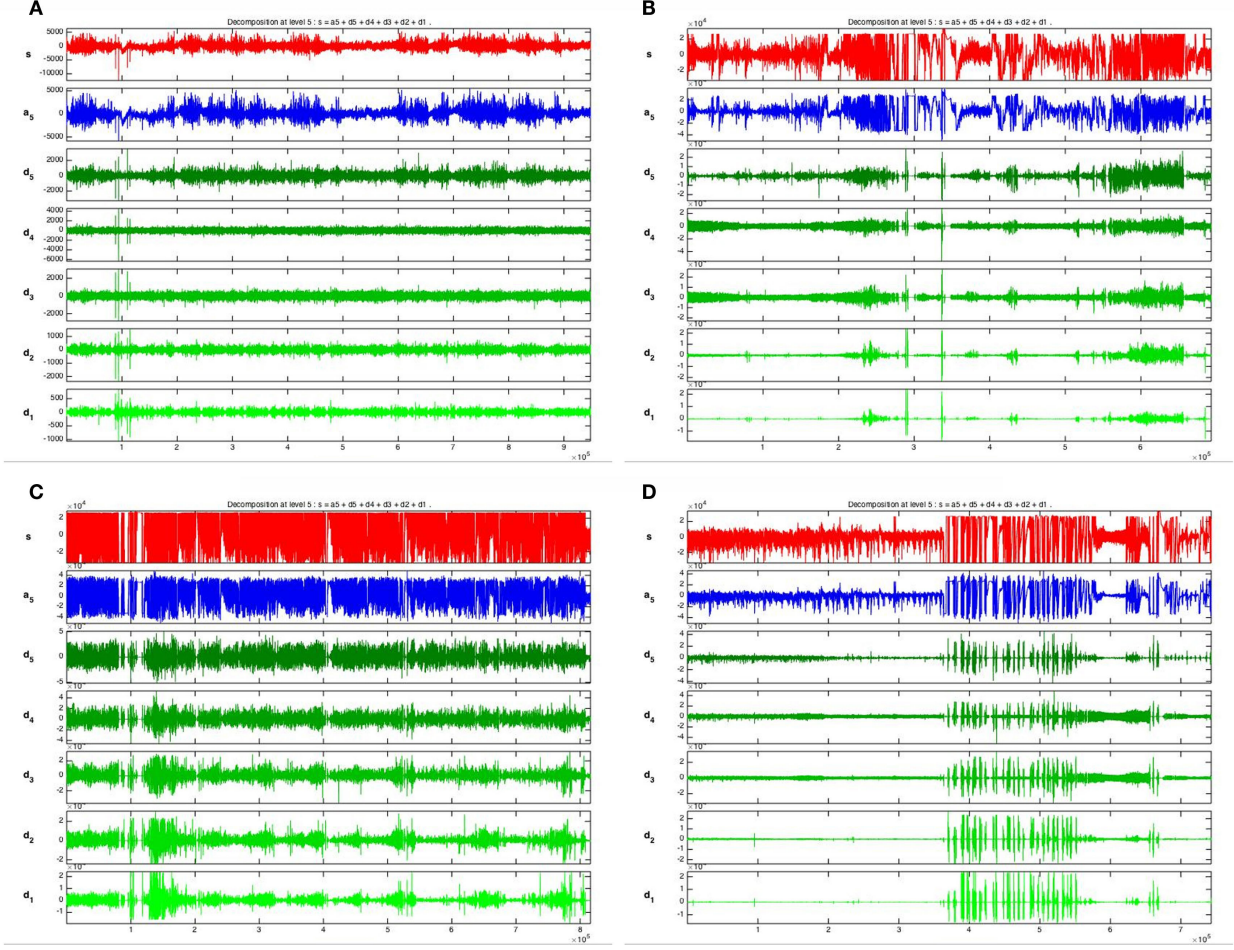
**Wavelet analysis of 10 min EEG in left DG**. The EEG signal in the left DG was decomposed into five layers and five frequency bands were extracted, spanning 16.5–31.25, 31.25–62.5, 62.5–125, 125–250, and 250–500 Hz [(fs/2)/2*^n^*]. **(A)** 1 day before SE; **(B)** 10 min before SE; **(C)** 10 min after SE; **(D)** 1 h after diazepam injection.

**Figure 2:**
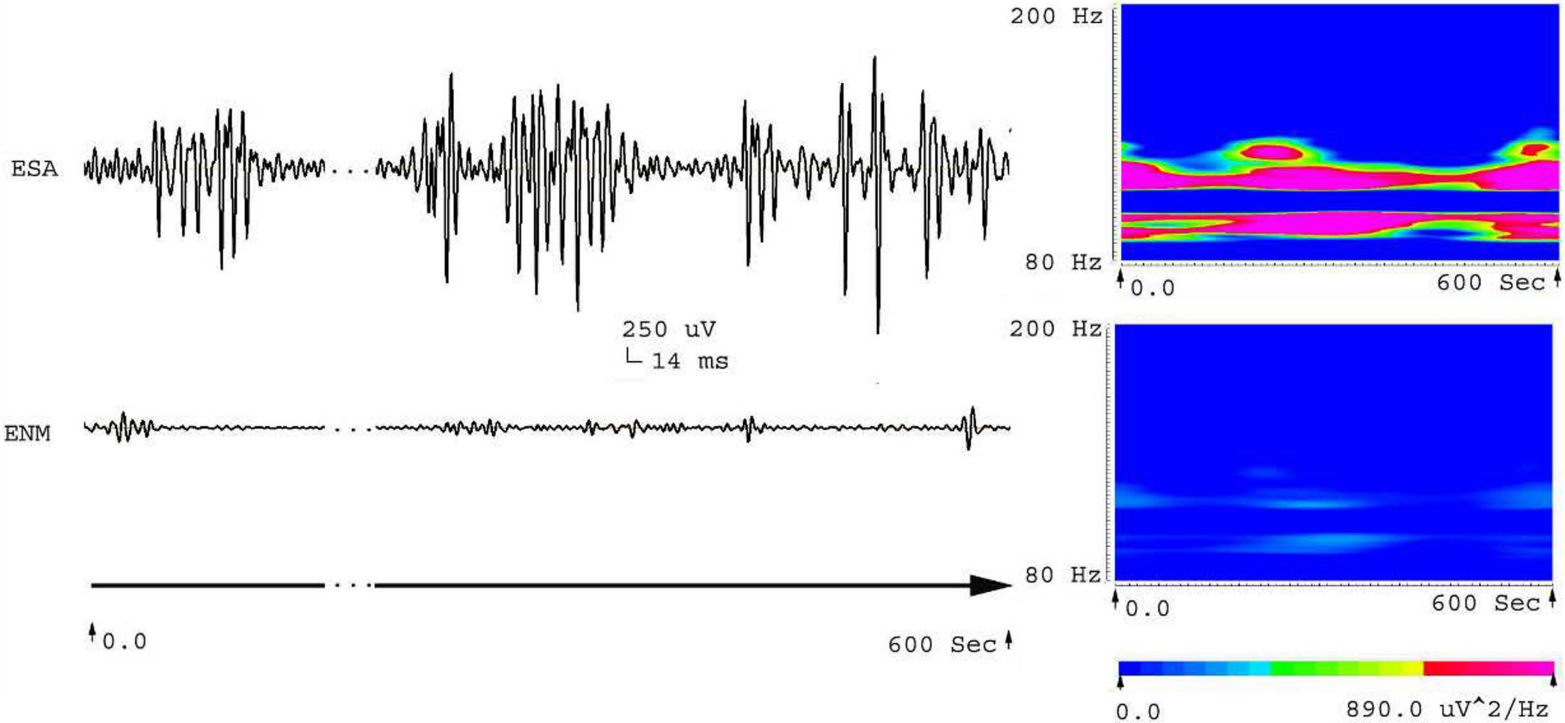
**Waveforms and spectrograms showing the spectral and temporal characteristics of HFOs at 80–200 Hz (ripple)**. The left waveforms are filtered with a band pass filter of 80–200 Hz. The right spectrograms are the accumulated time–frequency representations of the corresponding waveforms. ESA indicates an electrical signal from the CA1, which has epileptic activity; ENM indicates an electrical signal from a normal CA1 area. Both waveforms and spectrograms indicate that epileptic seizures were associated with increases in ripples. Those ripples were characterized by rhythmic bursts, which are significantly different from systematic artifacts such as power line noise and its harmonics. The spectrograms indicates that ripples occurred in two frequency bands around 90 and 110 Hz.

**Figure 3:**
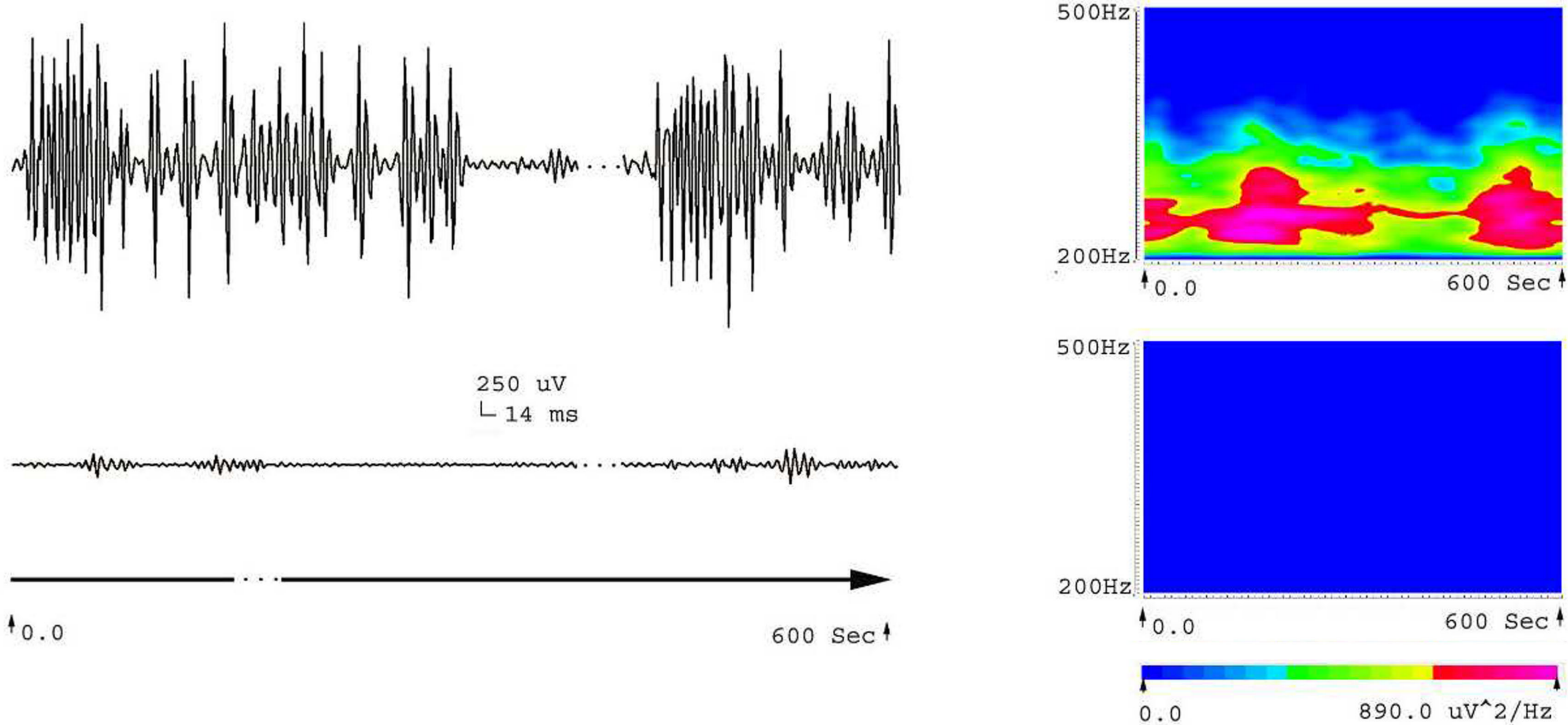
**Waveforms and spectrograms showing the spectral and temporal characteristics of HFOs at 200–500 Hz (fast ripple)**. The left waveforms are filtered with a band pass filter of 200–500 Hz. The right spectrograms are the accumulated time–frequency representations of the corresponding waveforms. ESA indicates an electrical signal from the area that has epileptic activity; ENM indicates an electrical signal from a normal area (control). Both waveforms and spectrograms indicated that epileptic seizures were associated with increases in fast ripples. The fast ripples are characterized by rhythmic bursts, which are significantly different from systematic artifacts, such as power line noise and its harmonics. The spectrograms indicate that the fast ripples occur around 220–260 Hz.

**Figure 4:**
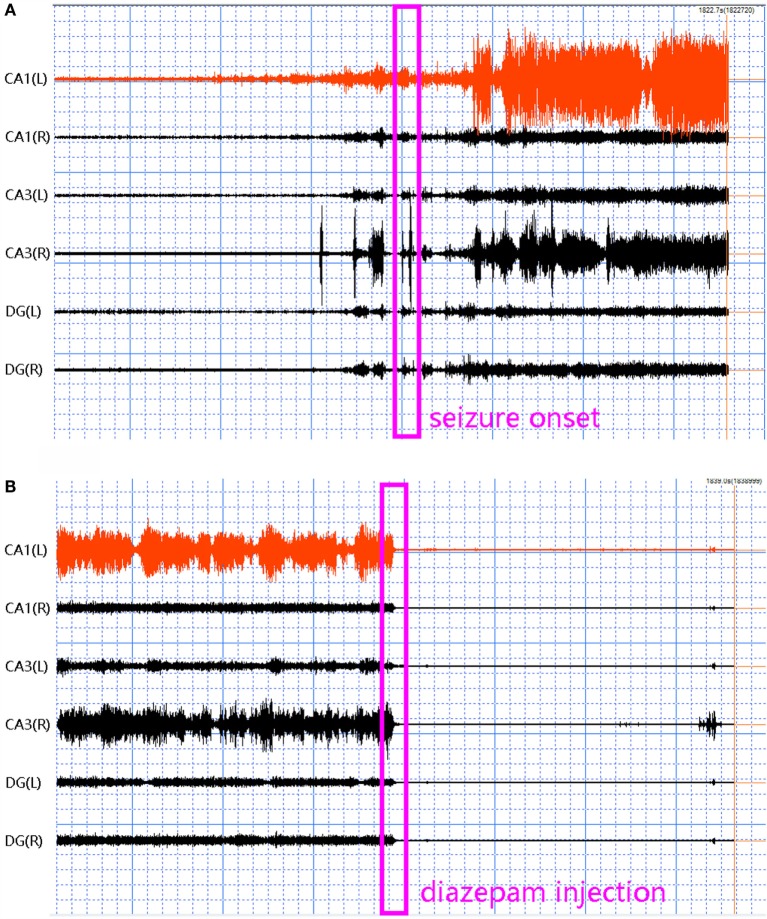
**Fast Ripples (FRs) in 30 min EEG at different states in the acute phase of epilepsy**. Paper speed: 0.00048 mm/s, sensitivity: 0.005 uv/mm. EEG was subjected to filtering analysis. **(A)** In the 15 min before and after SC, FRs increased gradually prior to stage 4 of Racine’s criterion. **(B)** In the 15 min before and after diazepam injection, FRs decreased gradually following diazepam treatment.

**Figure 5:**
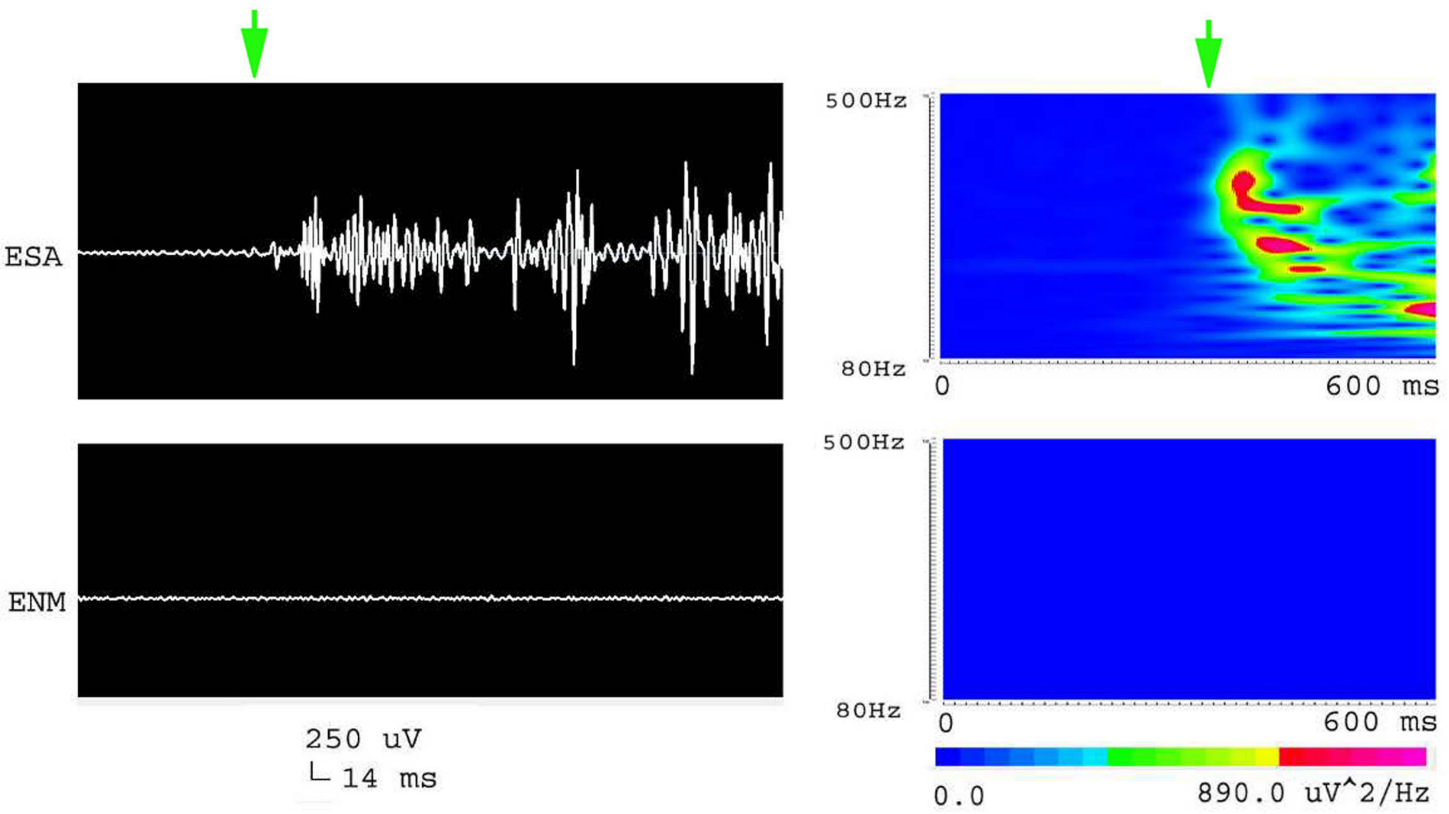
**Waveforms and spectrograms showing the spectral and temporal characteristics of the initiation of an epileptic seizure**. The left waveforms are filtered with a band pass filter of 80–500 Hz. The right spectrograms are time–frequency representations of the corresponding waveforms. ESA indicates an electrical signal from the CA1, which has epileptic activity; while ENM indicates an electrical signal from a normal CA1 area. The green arrows indicate the initiation of the seizure. Both waveforms and spectrograms indicated that the initiation of epileptic seizures were associated with increased high frequency oscillations at 80–500 Hz.

**Figure 6:**
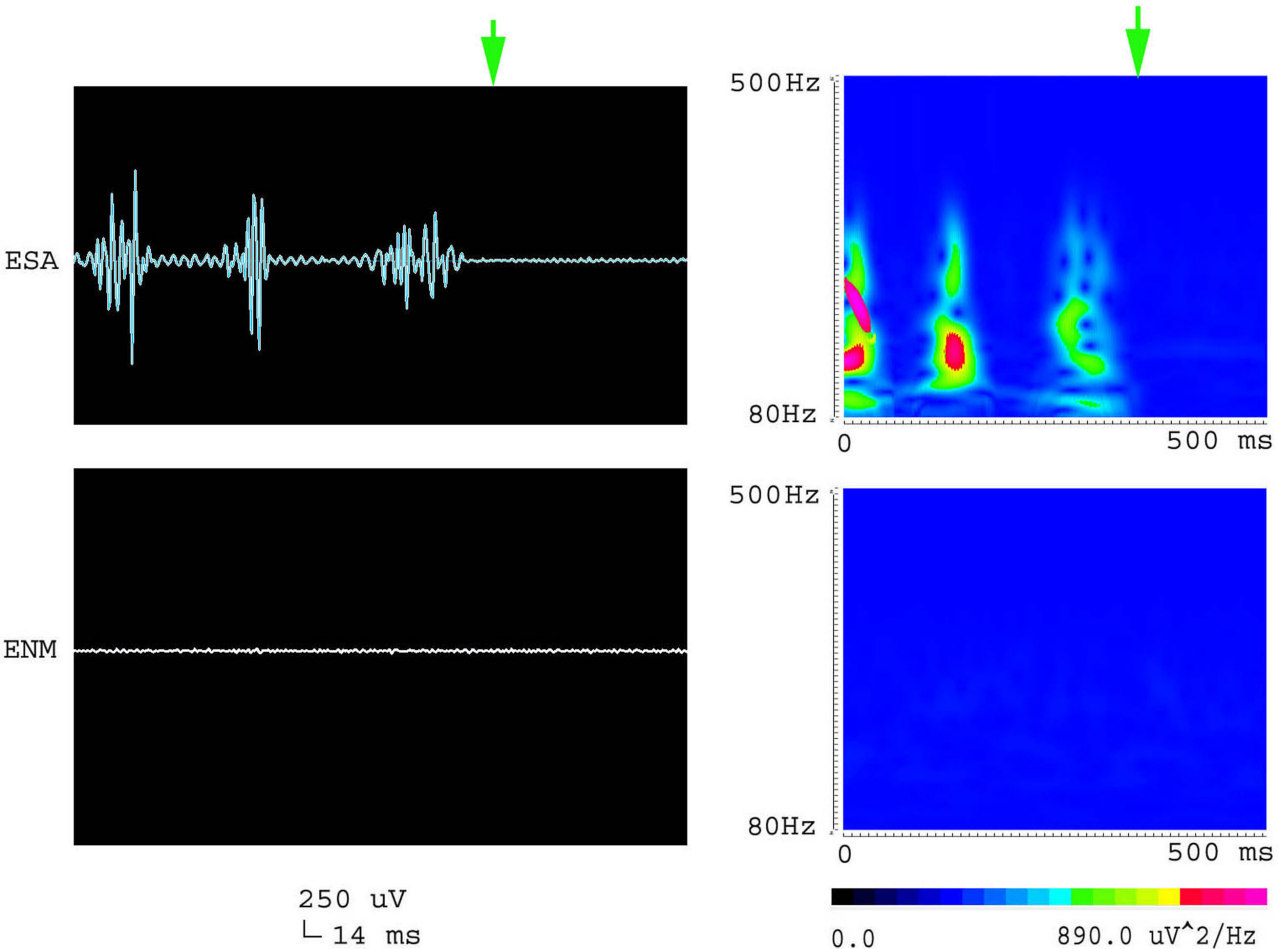
**Waveforms and spectrograms showing the spectral and temporal characteristics of the termination of an epileptic seizure**. The left waveforms are filtered with a band pass filter of 80–500 Hz. The right spectrograms are time–frequency representations of the corresponding waveforms. ESA indicates an electrical signal from the epileptic area, while ENM indicates an electrical signal from the normal area (control). The green arrows indicate the end of the epileptic seizure. Both waveforms and spectrograms indicated that the termination of epileptic seizures were associated with diminished high frequency oscillations at 80–500 Hz.

Five frequency bands, including HFO bands of 62.5–125 Hz, 125–250 Hz (ripple), and 250–500 Hz (FR), were identified in the CA1, CA3, and DG regions of normal hippocampus. (Figures 1A–D show the wavelet analysis of 10 min EEG in the left DG.) Waveforms and spectrograms showed the spectral and temporal characteristics of HFOs in ripples and FRs (Figures 2 and 3). After pilocarpine injection, HFOs increased gradually prior to stage 4 of Racine’s criterion (20); the peak was reached before injecting diazepam. HFOs expression decreased after injecting diazepam (Figures 4A,B, 5, and 6).

### Quantitative Analysis of HFOs

#### Acute Epilepsy

**Figure 7:**
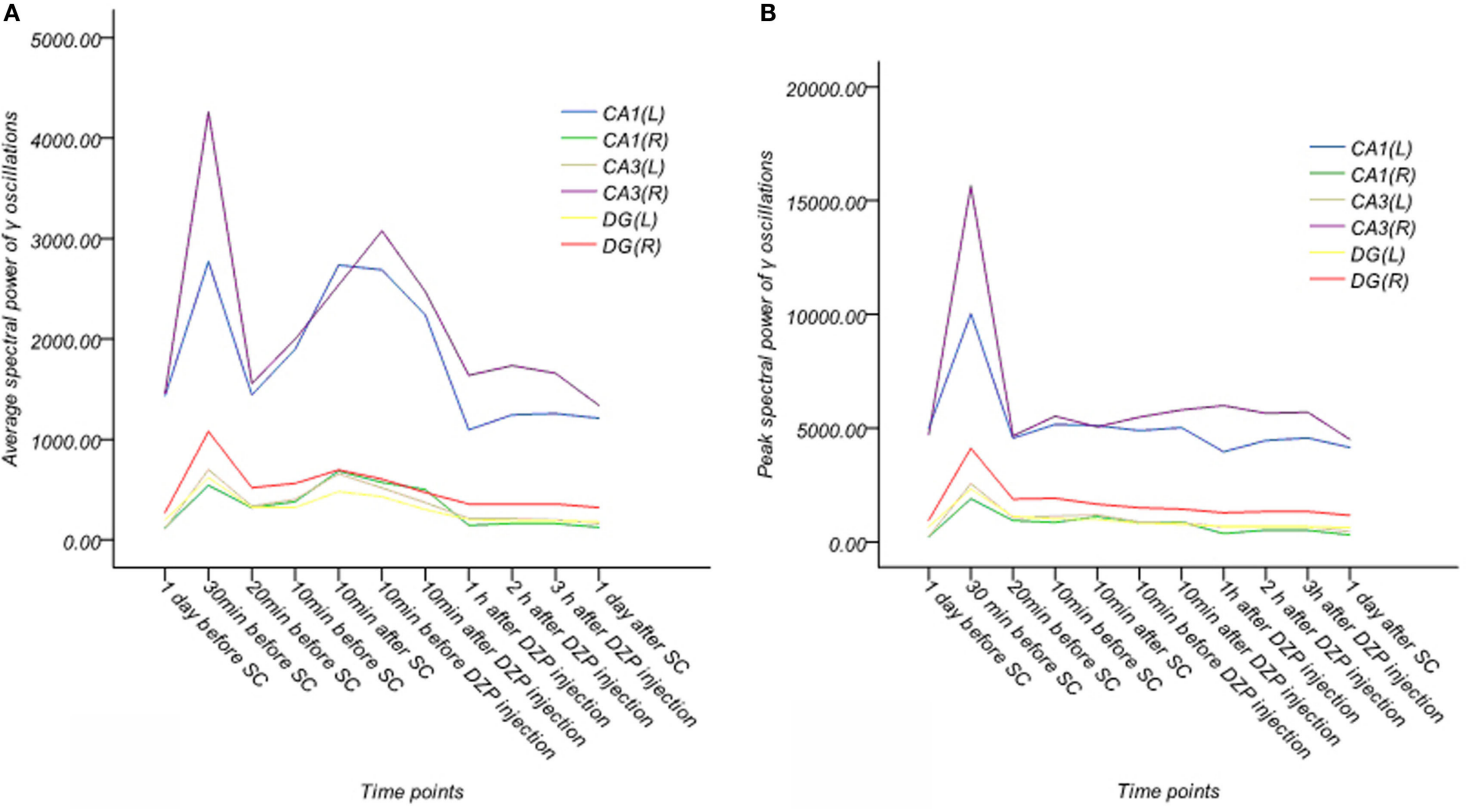
**Dynamic changes of average and peak spectral power of γ oscillations for six channels in the acute phase in one rat (A,B)**. **(A)** Average spectral power of γ oscillations. **(B)** Peak spectral power of γ oscillations. Average and peak spectral power of γ oscillations showed no systematic changes.

**Figure 8:**
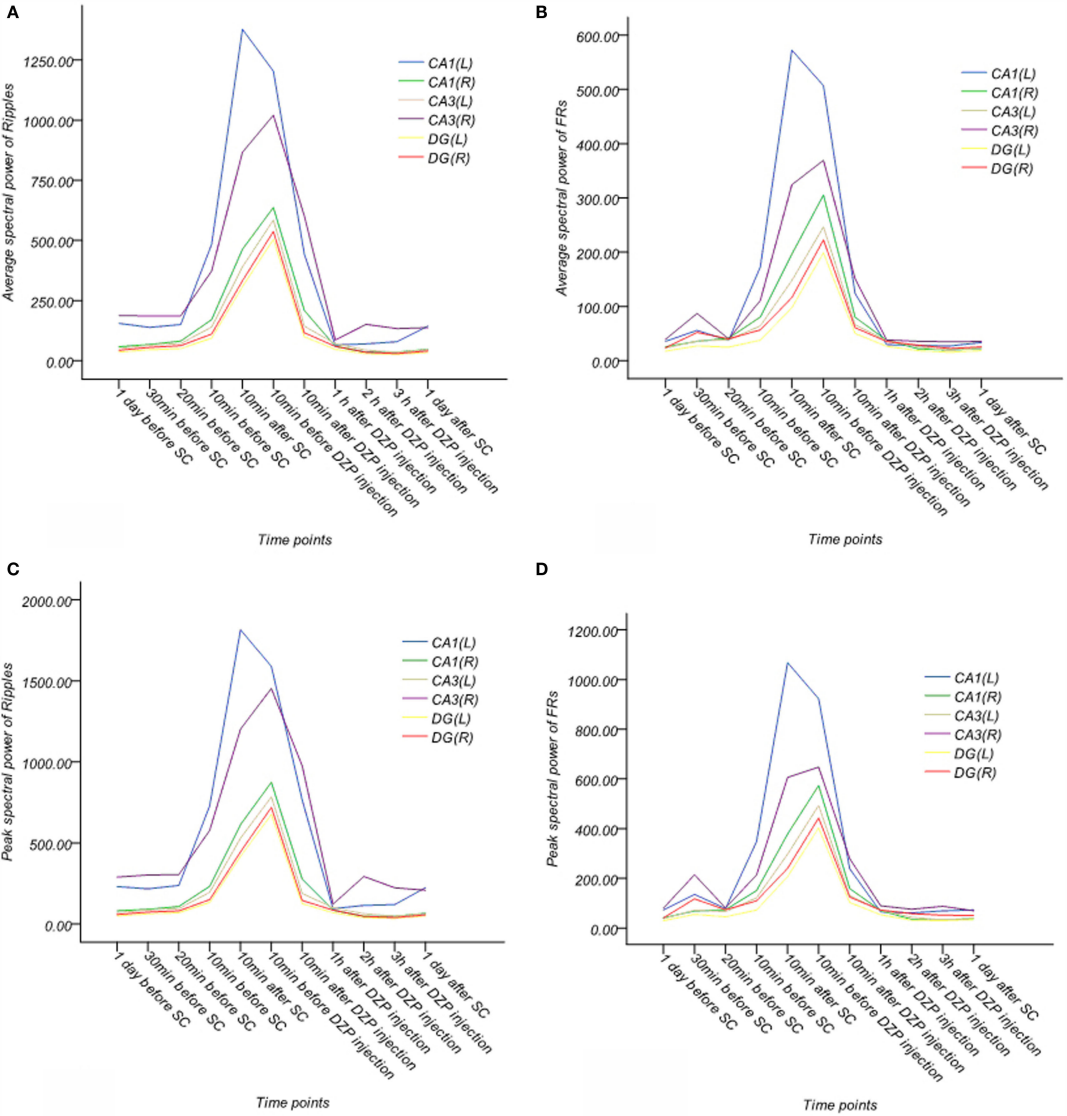
**Dynamic changes of average and peak spectral power of γ oscillations for six channels in the acute phase in one rat (A–D)**. **(A)** Average spectral power of ripples. **(B)** Average spectral power of FRs. **(C)** Peak spectral power of ripples. **(D)** Peak spectral power of FRs. The frequency bands of ripples and FRs exhibited specific and sharp increases, when SE began and then continuously decreased, finally ending at a lower level after injection of diazepam. EEG signals above 80 Hz appeared to be more stable than γ oscillations.

The average spectral power of γ oscillations showed no systematic changes (Figures 7A,B). While the frequency bands of ripples and FRs exhibited specific and sharp increases, when SE began and then continuously decreased, finally ending at a lower level after injection of diazepam (Figures 8A–D; Table 1). According to our data, the most significant dynamic changes in spectral power of HFOs were seen in the CA1 and CA3 regions (six rats) and in the DG (three rats). The leads where changes in HFOs spectral power were the most significant were defined as responsibility leads (RLs) (24). The spectral power changes of RLs maintained consistency between ripples and FRs. Peak spectral power exhibited similar dynamic changes as average spectral power.

**Table 1:** **Dynamic changes in the average and peak spectral power of HFOs recorded in RLs during the acute phase of epileptic model at different time points after SE (*n* = 15)**.

Time points	Average spectral power of ripples	Average spectral power of FRs	Peak spectral power of ripples	Peak spectral power of FRs
1 day before SE	89.49 ± 42.459	39.36 ± 19.705	163.49 ± 89.101	89.62 ± 67.735
30 min before SE	141.10 ± 61.508	71.67 ± 35.260^▲^	208.21 ± 115.592	133.81 ± 72.361^▲^
20 min before SE	128.37 ± 36.598^▲^	67.82 ± 29.181^▲^	194.19 ± 72.041	128.03 ± 61.828
10 min before SE	237.31 ± 181.316^▲^	105.75 ± 53.604^▲^	335.48 ± 260.026^▲^	205.84 ± 115.269^▲^
10 min after SE	575.18 ± 517.621^▲^	222.90 ± 150.658^▲^	797.98 ± 727.658^▲^	431.57 ± 312.644^▲^
10 min before diazepam injection	647.69 ± 357.794^▲^	278.50 ± 142.189^▲^	908.18 ± 497.798^▲^	518.65 ± 261.243^▲^
10 min after diazepam injection	416.25 ± 409.370^▲^	176.83 ± 171.352^▲^	625.12 ± 634.111^▲^	327.37 ± 274.054^▲^
1 h after diazepam injection	119.42 ± 72.776	60.71 ± 40.049^▲^	187.90 ± 119.798	128.13 ± 90.973
2 h after diazepam injection	110.44 ± 149.181	51.86 ± 64.826	188.28 ± 267.243	103.42 ± 123.546
3 h after diazepam injection	63.21 ± 40.882	36.52 ± 25.315	112.35 ± 88.305	85.77 ± 76.058
1 day after SE	80.50 ± 40.953	33.32 ± 13.934	135.28 ± 83.914	72.38 ± 47.342

*SE, status epilepticus; HFOs, high-frequency oscillations; RLs, responsibility leads*.

*^▲^*p* < 0.05 compared to normal rats (1 day before SE)*.

A further statistic analysis of the average spectral power and peak spectral power changes was made (Table 1).

In looking at the average spectral power of ripples, we noted ripple spectral power increased 20 min before SE (*p* < 0.05) and peaked 10 min before injection of diazepam. It decreased 10 min after diazepam, but remained higher than normal (*p* < 0.05) until returning to baseline at 1 h after diazepam injection, this effect persisted for 1 day after SE.

The average spectral power of FRs increased 30 min before SE (*p* < 0.05, compared with normal rats) and peaked 10 min before injection of diazepam. Like the ripples, it also decreased 10 min after diazepam. However, similar to the ripples, the spectral power was higher than normal (*p* < 0.05). The averaged spectral power of FRs recovered to baseline by 2 h after injection of diazepam and persisted for 1 day after SE.

The peak spectral power of ripples increased 10 min before SE (*p* < 0.05, compared with normal rats) and peaked 10 min before injection of diazepam. It decreased 10 min after injection of diazepam, but was higher than baseline (*p* < 0.05). This returned to baseline at 1 h after injection of diazepam and this effect persisted until 1 day after SE.

The peak spectral power of FRs increased 30 min before SE (*p* < 0.05, compared with normal rats) and peaked 10 min before injection of diazepam. Spectral power decreased 10 min after diazepam, but did not return to baseline until 1 h after diazepam injection. This effect continued for 1 day after SE.

#### Chronic Epilepsy

In the latent phase (1–7 days after SE), average and peak spectral power showed no significant changes (Table 2). By contrast, in the chronic phase (7–28 days after SE), average and peak spectral power showed a gradual downward trend compared with normal rats, but these did not reach statistical significance at all time points. There were no significant differences when we compared average spectral power and peak spectral power of two frequency bands (ripples and FRs) at different time points (Table 2).

**Table 2:** **Dynamic changes in the average and peak spectral power of HFOs recorded in RLs during the latent and chronic phases of epileptic model at different time points after SE (*n* = 9)**.

Time points	Average spectral power of ripples	Average spectral power of FRs	Peak spectral power of ripples	Peak spectral power of FRs
1 day before SE	102.86 ± 48.22	34.22 ± 14.15	167.65 ± 92.06	66.92 ± 32.67
1 day after SE	92.45 ± 34.82	36.69 ± 13.10	157.04 ± 76.62	81.21 ± 48.05
3 days after SE	104.72 ± 65.03	34.49 ± 15.36	175.95 ± 118.56	73.69 ± 56.63
7 days after SE	104.72 ± 67.54	35.69 ± 18.31	191.56 ± 131.81	82.81 ± 68.51
14 days after SE	91.87 ± 66.46	30.67 ± 10.56	144.84 ± 103.07	60.52 ± 25.35
28 days after SE	89.52 ± 49.08	28.26 ± 6.89	135.05 ± 79.19	53.02 ± 15.96

*SE, status epilepticus; HFOs, high frequency oscillations; RLs, responsibility leads*.

## Discussion

In our study, nine rats exhibited SRSs 3–10 days after SE, which marked entry into the chronic phase without significant effect from trauma. We studied the acute and chronic phases to investigate dynamic changes in spectral power of HFOs at different stages of epileptogenesis, so as to provide a theoretical basis for further quantification of epileptic activity.

High frequency oscillations s can be classified into normal and pathologic HFOs (pHFOs) (12, 25–27). Normal HFOs gradually mature with brain maturational processes (28). This reflects summated fast inhibitory postsynaptic potentials (IPSPs) in the somata of pyramidal cells, with the highest occurrence during SWS and the lowest occurrence during REM sleep (26). pHFOs are believed to reflect synchronized firing of abnormally bursting principal cells, the basis of spontaneous seizures (26, 27). pHFOs usually have much higher frequencies than normal HFOs. Above all, pHFOs occur mainly in regions of seizure onset where normal HFOs do not occur. Previous studies have indicated that HFOs of the normal hippocampus are γ oscillations or ripples, while FRs only occur in the epileptic hippocampus (12, 26). Since HFBS are weak (29), there are an increasing number of studies specifically focusing on the development of new methods of detection (21). We used new methods that provided greater sensitivity in detecting HFBS in an automated and quantitative way, rather than the more traditional and time-intensive visual inspection methods (21, 22). Our study found that FRs could occur in the normal hippocampus, although their amplitude or spectral power appeared to be stationary and significantly lower than the epileptic hippocampus. Parallel to previous studies (2, 23), dynamic changes in the spectral power of ripples and FRs were in agreement with the state of epilepsy, suggesting that the spectral power of HFOs may be utilized to distinguish between normal and pHFOs, rather than the frequency of HFOs.

The pHFOs are closely related to the seizure onset zone (SOZ) (7, 8, 11, 26). Patients undergoing complete resection of FR-generating tissue generally have a good surgical outcome (23, 30–32). Higher rates of HFOs were observed in the SOZ compared to other areas, particularly during interictal periods (33). The shorter the distance to the SOZ, the higher the frequency of the HFOs recorded (34). Spectral power analysis of HFOs have shown ripple oscillations (80–200 Hz) in the CA1 area (35), with the frequencies of intraburst spikes varying between 80 and 600 Hz. With increasing distance from the area of the burst generation, this activity appeared as HFOs (35). In our study, the spectral power of pHFOs was asymmetrically distributed between different sides and different regions of the hippocampus. Dynamic changes in spectral power of pHFOs in CA1 and CA3 were the most significant. Previous studies have shown that models of TLE are characterized by extensive neuronal loss, reactive gliosis, mossy fiber sprouting, granule cell dispersion, and synaptic remodeling in CA1 and CA3 (36, 37), which are the vulnerable regions after SE (38). It is assumed that these pathological changes lead to the genesis of new hyperexcitable circuits that generate SRS, characterizing the chronic phase. The consistency between electrophysiology and pathology indicated that significant dynamic changes in spectral power of pHFOs could be closely linked to SOZ. Regions where the spectral power of pHFOs varied most significantly may be responsible for SOZ.

The spectral power of HFOs can increase several minutes before seizure onset (26, 27). Compared with ripples, FRs may be a more sensitive and specific marker for epilepsy (39, 40). Our data demonstrated that low frequency EEG signals (<80 Hz) varied among animals, probably resulting from external noise, such as muscle artifacts and electrical noise. However, EEG signals above 80 Hz appeared to be stable. In particular, changes in EEG signals above 200 Hz were consistently identifiable in all animals, possibly because they were less affected by external noise. The average spectral power (over the entire time window) and the peak spectral power (spectral burst at a time point within the entire time window) of HFOs (>80 Hz) during ictal period were closely related to the occurrence of seizure. Both the average and peak spectral power of HFOs showed an increase of FRs at 30 min before SE (*p* < 0.05), indicating that FRs were more sensitive than ripples for quantifying dynamic changes in spectral power of pHFOs. It seems that the ictal average and peak spectral power of FRs are two parameters for predicting acute epileptic seizures, which could be used as a new quantitative biomarker and early warning marker of seizure.

Previous studies (8, 41, 42) have indicated that the spatial distribution and frequency of interictal HFOs are closely linked to epileptogenesis. The rates of interictal spikes and HFOs are higher inside the SOZ (43). However, HFOs are more specific and accurate than spikes for delineating the SOZ (43). We analyzed the spectral power of interictal EEG data, which was recorded from epileptic rats between seizures. The number of SRSs in chronic epileptic animals was 56.22 ± 12.15 within 4 weeks. However, compared with normal rats, neither the average spectral power nor the peak spectral power of ripples or FRs reached statistical significance. Therefore, there may be no relationship between interictal HFOs and seizure recurrence, contrary to previous findings (16, 44).

The formation of learning, memory, conditioned reflex, and other advanced neural activity has been suggested to mainly occur in the hippocampus, and HFOs are the foundation of these processes (12, 45). Normal HFOs are believed to play a critical role in cognition, including in information processing and consolidation of memory, sensory perception, and other advanced functions of brain (12, 26, 27). γ oscillations and ripples are widely believed to correlate with the formation of cognition (12). Frequent seizures can lead to severe cognitive deficits (46–48). Spatial memory deficits in the pilocarpine model of TLE have already begun during the latent period (47, 48) and cognitive deficits persist when animals had chronic spontaneous seizures. We found that average spectral power and peak spectral power of both ripples and FRs showed a gradual downward trend compared with normal rats during chronic phase. We hypothesize that changes in interictal HFOs spectral power in the hippocampus during the chronic phase of TLE may be related to cognitive deficits. However, this hypothesis needs further study.

## Ethical Publication Statement

We confirm that we have read the Journal’s position on issues involved in ethical publication and affirm that this report is consistent with those guidelines.

## Author Contributions

P-PS and JX made acquisition and interpretation of data and were involved in drafting the manuscript; they contributed equally to this work. YH conceived of the study, participated in its design, and helped to draft the manuscript. H-SC and B-KL participated in the design of the study and performed the statistical analysis. LJ revised it critically for important intellectual content. All the authors read and approved the final manuscript.

## Conflict of Interest Statement

The authors declare that the research was conducted in the absence of any commercial or financial relationships that could be construed as a potential conflict of interest. The reviewer RK and handling editor declared their shared affiliation, and the handling editor states that the process nevertheless met the standards of a fair and objective review.

## Abbreviations

EEG, electroencephalography; FR, fast ripples; HFBS, high frequency brain signals; HFOs, high frequency oscillations; MFBS, median frequency brain signals; pHFOs, pathologic HFOs; SC, status convulsion; SOZ, seizure onset zone; SRSs, spontaneous recurrent seizures; WDS, wet dog shake.
